# Improving nutritional status of children with Cerebral palsy: a qualitative study of caregiver experiences and community‐based training in Ghana

**DOI:** 10.1002/fsn3.788

**Published:** 2018-11-15

**Authors:** Claudia Mary Donkor, Jackie Lee, Natasha Lelijveld, Melanie Adams, Marjolein Meande Baltussen, Gifty Gyamah Nyante, Marko Kerac, Sarah Polack, Maria Zuurmond

**Affiliations:** ^1^ International Centre for Evidence in Disability London School of Hygiene and Tropical Medicine London UK; ^2^ Rabito Health Services Tema Ghana; ^3^ Department of Population Health London School of Hygiene and Tropical Medicine London UK; ^4^ No Wasted Lives Action Against Hunger UK; ^5^ Independent Consultant in Disability and International Development; ^6^ CBM International Bensheim Germany; ^7^ School of Biomedical and Allied Health Sciences College of Health Sciences University of Ghana Accra Ghana; ^8^ MARCH Centre Faculty EPH London School of Hygiene and Tropical Medicine London UK

**Keywords:** cerebral palsy, disability, feeding, Ghana, malnutrition

## Abstract

**Background:**

Cerebral palsy (CP) is the most common childhood disability worldwide, and evidence shows that children with CP are at an increased risk of malnutrition due to feeding difficulties. This qualitative study explores caregiver experiences of feeding before and after a community‐based training program in Ghana.

**Methods:**

Thirteen caregivers of children with CP, who were severely undernourished, were interviewed at the start of the training program. Eleven of these were interviewed again after a year of monthly group trainings and home visits, which included guidance on feeding. Four additional caregivers were interviewed at end line. Interviews explored caregivers’ mealtime experiences, as well as a 24‐hr dietary recall and a structured feeding observation checklist. Children's nutritional status was assessed by anthropometry.

**Results:**

Caregivers found mealtimes stressful due to time demands, messiness, and the pressure of providing enough quality food. They felt that the training program had helped reduced this stress and dietary recall data suggested some improved dietary quality. However, there was neither improvement nor deterioration in anthropometric status of the children.

**Conclusion:**

Group trainings were welcomed by caregivers and notably reduced stress around feeding times. However, future work is needed in order to improve anthropometric outcomes, including, but not limited to, greater focus on nutritional requirements during caregiver training interventions. Therapeutic feeding programs must also be better utilized and need to be better equipped to care for this group of children, including deviating from standard admission and treatment protocols.

## 1 INTRODUCTION

Cerebral palsy (CP) is a group of permanent neurological disorders affecting postural and movement development, attributed to nonprogressive disturbances in the developing fetal or infant brain (Donald, Samia, Kakooza‐Mwesige, & Bearden, 2014). It is one of the most common childhood disabilities affecting between 1.5 and 4 children per 1000 live births globally (Division of Birth Defects and Developmental Disabilities 2017; Donald et al., 2014). CP is thought to be even more common and severe in low‐income countries due to lack of access to early intervention and obstetric, neonatal, and rehabilitation services (Commey & Richardson, 1984; Groce et al., 2014). Despite this, relatively little is known about CP in African countries; there is especially limited evidence on population‐specific caregiver experiences and the impact of interventions (Donald et al., 2014).

In addition to other comorbidities including epilepsy, and communication and behavior difficulties, neurological disability is associated with a greater risk of malnutrition (Eunson, 2012; Kerac et al., 2014). Greater nutrient requirements, increased nutrient loss, and decreased nutrient intake all contribute to this. In addition, malnutrition can exacerbate disability through reduced muscle strength, diminished immunity, and reduced cerebral development (Kerac et al., 2014). Prevalence of underweight (low weight‐for‐age) among children with CP was found to be 42% in Uganda and over 80% in Nigeria (Kakooza‐Mwesige, Tumwine, Eliasson, Namusoke, & Forssberg, 2015; Ogunlesi, Ogundeyi, Ogunfowora, & Olowu, 2008), far exceeding the 30% prevalence that the WHO defines as being “very high” (WHO 2015). Given the increased risk of mortality associated with malnutrition, as well as long‐term health implications, this issue needs to be urgently addressed.

As children with CP commonly have difficulties controlling their oral and facial muscles to chew and swallow, ensuring their adequate nutritional intake, as well as preventing aspiration, can be difficult for caregivers (Adams et al., 2011; Mobarak, Khan, Munir, Zaman, & McConachie, 2000). Few studies have explored in‐depth caregiver's experiences of feeding difficulties in children with CP and interventions to address this. The study presented in this paper is part of a wider research project in Ghana, which assessed feeding difficulties and nutritional status among 76 children with CP and explored the impact of a 12‐month, community‐based, parent training program. The training program was based on the “Getting to Know Cerebral Palsy” (GTKCP) manual (http://disabilitycentre.lshtm.ac.uk/getting-to-know-cerebral-palsy/) and comprised of 11 modules delivered in group support sessions, including one on “Eating and Drinking.” The program also included monthly home visits by training facilitators. Prior to implementation of the training, a baseline survey found that the prevalence of malnutrition was very high: 65% of children aged <5 years were categorized as underweight, 54% as stunted, and 58% as wasted. Reported difficulties with the child's feeding were common, and this was associated with the child being underweight (Odds Ratio 10.7 95% CI 2.3–49.6) and poorer caregiver quality of life (*p* < 0.001) (Polack et al., 2018; Zuurmond et al., 2018).

This article presents the findings of a qualitative study focussed on exploring caregivers’ experiences of feeding, before and after the Ghana community‐based training program was implemented, as well as anthropometric data and 24‐hr dietary recall.

## 2 METHODS

### 2.1 Study design

This qualitative study was nested within a larger before/after study seeking to evaluate the impact of the community‐based parent CP training program: GTKCP. Findings from this wider evaluation are published separately (Polack et al., 2018; Zuurmond et al., 2018). This qualitative study used semistructured interviews with primary caregivers of children with CP, both prior to (“baseline”) and after 12 months of implementing the training manual (“end line”). In addition, data were collected using a 24‐hr dietary recall, a feeding observation checklist, and anthropometric assessment of the children's nutritional status. These are presented to add context to these qualitative findings.

### 2.2 Setting

The CP training program was implemented in eight geographical regions in Ghana. This qualitative study took place in four of those regions, selected based on feasibility of access: Agogo, Dodowa, Sunyani, and Techiman. The majority of interviews took place in participants’ homes (*n* = 14). However, some interviews were conducted at the Physiotherapy Unit at Agogo Hospital, at caregivers’ workplaces, or at a school. While efforts were made to ensure privacy, around half of the interviews had other children or adults present, with the interviewee's permission, due to the communal nature of home and work environments.

### 2.3 Ethical approval

Ethical approvals were obtained from Noguchi Memorial Institute for Medical Research in Ghana (NMIMR‐IRB CPN 053/14‐15) and The London School of Hygiene and Tropical Medicine (LSHTM ref: 8905;10147;10880). Informed written or thumb‐printed consent was obtained from each participant in advance of the research. Any child identified as wasted at baseline was referred to local therapeutic feeding programs.

### 2.4 Research team and reflexivity

Interviews were conducted by CD who is a female, Ghanaian physician, fluent in both English and Twi languages and JL, an Asian Australian physiotherapist. Data analyses were conducted by CD, JL, and NL.

CD was introduced to participants at baseline interviews as a doctor and researcher. Interviews were conducted in Twi and/or English, at the preference of the participant. Both CD and JL reflected on their occupational and cultural perceptions during daily reflective diary entries and during interpretation of interviewees’ responses.

### 2.5 Sampling

Participants were selected from caregivers of children (ages 1 to 16 years) with CP registered in a community‐based rehabilitation program, who were invited to participate in the parent training program. A total of 15 caregivers of children who were identified, through anthropometric assessment (described below), as being moderately or severely malnourished were purposively selected for qualitative interview. No exclusions were made on severity of CP. Participants were invited to participate by phone or by home visit. In total, 13 of the 15 caregivers identified were available for interview.

The end‐line sample consisted of 11 (out of the 13) caregivers from the baseline sample and four additional caregivers who were identified as having undernourished children during the course of the training program. In total, 16 children with CP were included as one caregiver was taking care of two children with CP. Two caregivers from the baseline sample were excluded, as one did not take part in the training program and the other was unavailable due to the hospitalization of her child. The end‐line interviews were conducted 12 months after the baseline interviews, ensuring similar seasonal food availability (July 2015 and July 2016, respectively).

### 2.6 Data collection

A semistructured interview guide was developed based on areas covered by the training program and included questions on positioning, utensils, and feeding improvement strategies. The end‐line topic guide was similar to the baseline with additional questions about the perceived impact of the training on feeding. Interviews lasted approximately 40 min and were audio‐recorded and later transcribed into English.

A 24‐hr dietary recall questionnaire and a feeding observation were also conducted. The 24‐hr dietary recall recorded feeding frequency, types of food, and food modification methods. The feeding observation used a structured observation checklist during observation of one meal. The interview guide, food recall questionnaire, and observation checklist were all based on tools developed for a study by Adams et al. in Bangladesh (Adams, 2009).

Child anthropometry was conducted as part of the wider research on all children included in the training program. Where possible, standing height was measured for children > 5 years and recumbent length was taken for children <5 years, both measured to nearest 0.1 cm. Knee height was recorded for all children to the nearest 0.1 cm using anthropometric calipers (CLPR65, MediForm, USA) (Froehlich‐Grobe, Nary, Van Sciver, Lee, & Little, 2011). Mid‐upper arm circumference (MUAC) was recorded for all children aged 6–59 months (MUAC tape, Teaching Aids at Low Cost). Weight was recorded to the nearest 0.1 kg. If it was not possible to measure a child's height or recumbent length, knee height was used as a proxy measure, based on the line of best fit on a scatter graph for children with complete data. Weight‐for‐age (WAZ) (children <10 years only), height‐for‐age (HAZ), and weight‐for‐height (WHZ) (children ≤5 years only) *z*‐scores were calculated based on WHO growth standards, using WHO Anthroplus software. As per standard case definitions, children with z‐scores ≤ −2 were defined as stunted (HAZ), wasted (WHZ), and underweight (WAZ), respectively. Children with MUAC <125 mm were also defined as wasted (WHO 2015).

The severity of each child's CP was assessed using the Gross Motor Function Classification System (GMFCS). Based on this system, scores one and two are defined as mild, three is moderate, and four and five are classified as severe CP (Rosenbaum, Palisano, Bartlett, Galuppi, & Russell, 2008).

### 2.7 Analysis

A thematic analysis was conducted on the interview data; themes were discussed with coauthors, and key themes were triangulated with structured observations, the 24‐hr dietary recall, and anthropometric data.

All the data were transcribed by CD and coded by CD, JL, and NL. Themes were derived both inductively and through predetermined areas of discussion, based on known feeding challenges from the literature, using NVivo 10 software. See Figure S1 for coding tree.

## 3 RESULTS

### 3.1 Sample description

Table 1 presents demographic characteristics of the caregivers and their children. A summary of the children's nutritional status is presented in Table 2 (individual data are presented in Table S1). The average age of the children at baseline was four years two months, ranging from 1 year 5 months to 11 years 11 months. The sample included 8 males and 11 females; although a range of CP severities was represented, the sample included mainly moderate‐to‐severe cerebral palsy. All caregivers were female except one; the majority were mothers, and they were all aged above 20 years.

**Table 1:** Basic demographics of caregivers and children interviewed at baseline and/or end line

ID number	Caregiver relationship	Caregiver age (years)	Caregiver occupation	Child sex	Child age (baseline)	CP severity (GMFCS)	Time of interview
C1	Grandmother	40–60	Unemployed	Male	3 years 1 month	5	Baseline and end line
C2	Uncle	40–60	Unknown	Female	11 years 11 months	1	Baseline only
C3	Mother	30–40	Unemployed	Male	4 years 3 m	4	Baseline and end line
C4	Mother	30–40	Unemployed	Female	5 years 3 months	5	Baseline and end line
C5	Mother	40–60	Unemployed	Male	8 years 10 months	5	Baseline and end line
C6	Aunt	30–40	Unemployed	Female	1 year 5 months	4	Baseline and end line
C7	Mother	30–40	Employee	Male	2 years 6 months	5	Baseline and end line
C8	Grandmother	40–60	Unemployed	Female	5 years 1 month	3	Baseline only
C9	Mother	20–30	Employee	Female	3 years 6 months	4	Baseline and end line
C10	Mother	30–40	Farmer	Male	4 years 6 months	5	Baseline and end line
C11	Mother	30–40	Farmer	Female (a)	1 year 6 months	4	Baseline and end line
				Female (b)	6 years 4 months	1	Baseline and end line
C12	Mother	20–30	Unknown	Male	2 years 3 months	3	Baseline and end line
C13	Mother	30–40	Employed	Female	1 year 7 months	2	Baseline and end line
C14	Mother	30–40	Unemployed	Female	4 years 5 months	2	End line only
C15	Mother	20–30	Employee	Male	2 years 6 months	3	End line only
C16	Mother	30–40	Employee	Male	2 years 6 months	3	End line only
C17	Mother	40–60	Unemployed	Female	4 years 8 months	4	End line only

CP: cerebral palsy; GMFCS: Gross Motor Function Classification System.

**Table 2:** Summary of the nutritional status of children with CP at the time of baseline and end‐line interviews

	Baseline (*n* = 18)	End line (*n* = 16)
Mean weight‐for‐height, z‐score	−2.47	−2.24
Mean height‐for‐age, *z*‐score	−2.13	−2.62
Mean MUAC (mm)	151	149
% Wasted	50%	50%
% Stunted	50%	50%

MUAC: mid‐upper arm circumference; Stunted: height‐for‐age z‐score ≤ −2; Wasted: weight‐for‐height *z*‐score ≤ −2 or MUAC<125.

### 3.2 Structured observation of feeding

A short feeding observation was conducted for 11 children at baseline and 10 of the same children at end line, plus three additional children. Some form of oral‐motor dysfunction was present in all the children in the sample; symptoms, either observed during the research or mentioned by caregivers, included difficulties in chewing and swallowing, presence of vomiting, drooling, choking, and coughing.

At baseline, one child was observed to self‐feed and nine children were upright and supported during feeding. At end line, seven children were observed to self‐feed (or partially self‐feed) and eight children were upright and supported during feeding. The other children were fed reclined in the caregiver's lap. During baseline observation, it was noted that poor positioning often resulted in the oral spillage of fluids and food. Observations at end line demonstrated that positioning had improved, but one third of caregivers found it difficult to position their children, especially those with severe CP. Despite caregivers’ efforts to position their child's body upright, the neck often remained extended.

At baseline, caregiver frustration during mealtime was commonly observed, manifesting as force‐feeding in some instances. During end‐line observations, none of the caregivers appeared frustrated or upset with their child; most were conversational or attentive to their child's eating and drinking.

### 3.3 24‐hr dietary recall

All caregivers were interviewed about their child's diet in the past 24 hr. Children were largely fed cereal‐based porridge and some vegetables (Table 3). Despite most caregivers identifying fruit and protein sources (eggs/meat/fish) as healthy foods during the in‐depth interviews, few provided these to their child with CP within the past 24 hr. Fish was the most common source of protein; however, many children had inadequate or no protein in the diet, based on the food recall data. This was particularly so at baseline, with only 5/13 reporting any consumption of protein, while the end‐line survey suggested some improvement in protein intake (*n* = 12 consuming one or multiple sources).

**Table 3:** Results of 24‐hr dietary recall at baseline and end line

Food group (consumed in the past 24 hr)	Baseline		End line	
	*n*	%	*N*	%
Cereals and grains	13	100	16	100
Root vegetables	2	15	0	0
Vegetables	7	54	12	75
Fruit	0	0	0	0
Legumes	2	15	3	19
Eggs	1	8	5	31
Dairy	0	0	3	19
Nuts	1	8	7	44
Fish	4	31	8	50
Meat	0	0	1	6
Sugars	3	23	6	38
Breast milk	2	15	0	0
Vitamin supplements	0	0	1	6
Total sample	13	100	16	100

### 3.4 Semistructured interviews

Findings from the interviews are divided into pre‐ and posttraining. Several recurring themes have been identified, including length of mealtimes; modification of food; self‐feeding; quality and quantity of food; and caregiver stress.

### 3.5 Mealtimes at pretraining

#### 3.5.1 Length of mealtimes

This varied from 10 min to 3 hr. While there were examples of children feeding quickly, especially when the child liked the food, in many cases, long meal times were the norm, and some further lengthened by other comorbidities. It was reported by the mother of a 5‐year‐old girl that feeding was often prolonged due to recurrent seizures.

It takes three minutes for her to finish chewing her food. If you speed up the mealtime, she coughs and chokes. One needs time and breaks in between. (referring to how long it takes to chew each morsel of food given) (C9)

#### 3.5.2 Modification of food

The majority of caregivers reported the need to modify the consistency of the food, making them softer or liquidized, to enable feeding. Others described preparing meals without pepper for their child as Ghanaian food typically has high levels of spice. Some caregivers reported giving the child only what he/she wanted to eat/drink to make feeding easier.

He cannot chew meat or fish so I have to mash it into a powdery form before I add it to the food he is going to eat. (C3)

I have to make the palm‐nut soup thicker and make the rice softer before I feed her. (C4)

#### 3.5.3 Self‐feeding

All but one of the carers perceived that their child was unable to self‐feed. This was for a variety of reasons, including inability to use cutlery, messiness, and requiring positioning support.

He is unable to eat like the other children so I have to feed him like a baby. (C3)

He cannot eat by himself. He cannot hold cutlery so he needs to be fed. (C1)

Conversely, force‐feeding was also deemed necessary by just over half of the caregivers at baseline (8/13). Some felt that this was required to ensure a nutritious or balanced diet; some felt that this was the only way to get their child to eat at all.

When she is fed, the food has to be forced past her teeth and teeth pushed together by holding her jaws closed…food has to be pushed down her throat. (C4)

#### 3.5.4 Quality and quantity of food

Perceptions varied regarding the amount of food consumed. More than half of the respondents reported that their children had enough food at mealtimes. They also felt that they were able to provide some nutritious food each day. Conversely, half of participants felt their children needed more food, especially nutritious food.

Yes! [She finishes everything on her plate]. Often, it is not enough! (C2)

One needs patience when feeding him. If you look at his head‐shaking during mealtimes as a sign to stop, you will be deceived and he will not have enough food to eat. (Referring to child's involuntary head movements) (C12)

#### 3.5.5 Caregiver stress

More than half of respondents expressed frustration or negative feelings about mealtimes. Most were concerned by the time‐intensive and effort‐demanding nature of mealtimes, which prevented them from engaging in other activities, including housekeeping and paid work. Their comments suggest that feeding is often physically exhausting as well as stressful.

Taking care of her is stressful and strenuous. The whole day revolves around her and activities for her. By the time you go to bed, you are exhausted and have generalised body pains. You wake up exhausted too. (C9)

Since it takes such a long time to feed him, I often do not have the time to do other things. I am unable to leave him and even go and work because of his condition. I need to work and support him and the other children but I just cannot. This makes me very unhappy. (C10)

Caregiver stress was related to all the above‐identified feeding challenges, namely, the length of meal times, the need to modify food and prepare separate meals from those of the rest of the family, the perceived inability of children to feed themselves, the messiness caused during meals, and the pressure of providing enough and good quality food. Messiness was a particular concern for caregivers; it led to extra chores and was perceived as a factor in deterring others from helping caregivers with feeding.

Because he is my son, I have more patience for him although sometimes I get angry with him as well. Sometimes when I get angry, I beat him. (C3)

I am worried about the fact that he is not eating well in order to crawl and walk. I am unable to sleep at night from worrying about it. (C13)

It is also hard and tiring feeding him. Sometimes, he is messy and I get covered with his food. When he is being fed, he spurts out the food and some lands on my face. It is very tiresome feeding him. (C3)

### 3.6 Mealtimes posttraining

After attending the “Getting to Know Cerebral Palsy” program, caregivers reportedly felt less stressed about meal times and felt that the training had been helpful. Areas of the training particularly highlighted as helpful included positioning, self‐feeding assistance, and suggestions on improving the variety of food groups in their child's diet. Although observations noted that positioning was still a challenge, multiple caregivers felt that the training had been helpful with regard to positioning their child:

Initially [my daughter] was not able to eat or drink properly, but after the training there has been much improvement…Through the training we've understood what the condition is… we've learnt we need to be patient when giving [our children] the food, and also the types of food to give them… We've been able to apply [our] knowledge and things are much better C11a

When he tilts the head, I help him to bring it forward because [I've] been told that if I don't do that, the food will not go down well (C12)

There were also positive feelings expressed about being able to connect with other caregivers of children with CP during the training:

I felt encouraged, I was hopeful, because others would say ‘my child started walking at age 4 or 5, so don't worry, your child is not there yet’, so gradually, she will get to that age where she can walk. So I was very encouraged by their words (C13)

There was some evidence of improved dietary diversity based on the 24‐hr dietary recall questionnaire (Table 3). Some caregivers also explained that they were able to give more diverse foods after having been given advice on appropriate modifications:

At first he could only eat porridge and rice, but now he's able to eat all types of food. I have to modify some solids and for other foods like ampesi [boiled yam], I have to mash it a little bit before I give. (C5)

Despite some improvements and positive reflections from caregivers posttraining, caregivers still expressed a number of frustrations and concerns relating to feeding, and there was almost no evidence of improved nutritional status of the children based on anthropometry (Table 2).

#### 3.6.1 Length of mealtimes

Meal times still reportedly lasted between 10 min and 2 hr; therefore, some still being either too long or too short.

#### 3.6.2 Quality and quantity of food

The dietary recall data suggest some improvements in high‐protein foods such as peanuts, eggs, and fish. However, there was still a notable lack of fruit in the diet, despite almost all caregivers identifying fruit as healthy when asked to give examples of healthy foods.

Half of caregivers at end line perceived that their child with CP ate less than other children;

He doesn't eat the same quantity as other children his age and this is because of his condition (C10)

Some oral‐motor dysfunction such as vomiting and drooling was interpreted as the child being full or not wanting their food, resulting in premature completion of meal times:

Every time [I give] him porridge for breakfast, he starts spewing it out…That tells [me] that he's fed up with that meal (C3)

#### 3.6.3 Self‐feeding

Self‐feeding continued to be viewed quite negatively by caregivers, largely due to its association with mess. Even caregivers of children with moderate or mild CP identified self‐feeding as a challenge.

He'll put his hand in [the food] and just mess up the place. So [I] just [take my] time to do it. (C10)

#### 3.6.4 Caregiver stress

Although many caregivers reported reductions in mealtime stress, participants still voiced concerns about long mealtimes and financial strains. Even caregivers, who were relieved of some mealtime obligations by their child attending school, still felt anxious about whether their child was receiving adequate nutritional support during school hours.

Another source of concern was the lack of support received by caregivers from routine medical and nutrition services. The training intervention encouraged caregivers to seek nutrition support for acutely malnourished children. However, when visiting nutrition clinics, some were not referred for treatment, some were given advice that they felt was inappropriate, while others were referred for treatment but had difficulties attending appointments and did not receive follow‐up. This disconnect with nutrition services seemed to be contributing to the limited care‐seeking habits of caregivers:

The nutritionist said for the child to be eating Plumpy'Nut raw, instead of having it mixed up in the food now. But with that, he doesn't eat it … [The nutritionist] didn't ask why he wasn't eating it. I didn't ask [for advice] either. (C13)

## 4 DISCUSSION

This study identified six recurring themes which describe caregivers’ experiences of feeding their children with CP: lengthy mealtimes; the need to modify food consistency; children's inability to self‐feed; messiness at mealtimes; difficulty providing adequate quality and quantity of food; and caregiver stress, which was associated with all of the other experiences. These challenges mirror caregiver reports in previous studies (Adams et al., 2011; Sullivan et al., 2000).

After 12 months of the training program, caregivers felt that mealtimes were less stressful. Mealtimes can provide opportunities for caregivers and children to develop relationships. However, our baseline findings support studies in other settings; where for caregivers of children with CP, feeding is stressful or unenjoyable (Adams, 2009; Andrew & Sullivan, 2010; Gangil, Patwari, Aneja, Ahuja, & Anand, 2001). Furthermore, previous studies have found that stress can contribute to inappropriate feeding positions and methods, force‐feeding, and verbal and physical abuse (Adams et al., 2011). The reduction in stress felt by caregivers in this study is therefore an important achievement of the training program and reinforces the value of caregiver training, as evidenced in previous studies with similarly positive outcomes (Adams et al., 2011).

In the follow‐up feeding observations, the majority of children were supported upright during feeding and there was some small improvement in rates of self‐feeding. Furthermore, although the 24‐hr dietary recall found that the diet at baseline and end line consisted largely of cereal‐based foods and some vegetables, there was an increase in protein content for some children following the training. Ongoing difficulties with positioning, especially head positioning, were still observed posttraining. This may have been due to a lack of uptake of supportive seating and an overreliance on lap‐feeding. Caregivers, however, reported feeling better about positioning their child. They also reported feeling reassured by meeting other caregivers.

Despite the positive effects of the training program, it did not result in clear improvements in the unacceptably high prevalence of malnutrition in this group. While it is difficult to say how much of this reflects reduced disability‐associated growth potential (Krick, Murphy‐Miller, Zeger, & Weight, 1996), the magnitude of anthropometric deficit suggests that there is scope for further improved nutritional status in this group. Malnutrition is a known challenge for children with neuro‐disabilities and children with CP are more likely to die young because of malnutrition (Colver, Fairhurst, & Pharoah, 2013). It is known that feeding difficulties in this population limit the ability to take in sufficient nutrition. It is also known that many have increased nutrient requirements, especially when comorbidities, such as seizures, are present (Kerac et al., 2014). Aspiration is also common in children with CP, which can lead to secondary chest infections, again resulting in higher nutritional needs (Tomkins & Watson, 1989).

This combination of issues makes it very difficult to provide enough nutrients to children with CP. In light of this, it is important to acknowledge the stabilization, rather than deterioration in nutritional status of the children involved in this training intervention, as a positive outcome. Further nutritional benefits may have been achieved with a greater emphasis given to the feeding component of the whole training package, especially attitudes toward self‐feeding, for both direct caregivers and wider household members. A longer intervention may also have helped, given that it takes time for children to adapt to changes in feeding practices (Rosenbaum & Gorter, 2012). However, previous studies suggest that improving nutritional status in children with CP through caregiver training alone is a challenge. A similar training in Bangladesh also found minimal improvement in children's nutritional status as a result of caregiver training (Adams et al., 2011).

In high‐income countries, children with CP regularly receive additional nutritional support via oral and nonoral means. However, children in this study who were referred to as mainstream therapeutic feeding services still showed no improvement in nutritional status and caregivers reported feeling largely dissatisfied with the support they received. Effective interventions to improve the nutritional status of children with CP in a low‐income context, where children are relying on oral feeding alone, are urgently required. The nutrition sector needs to provide program staff with better guidance and training on how to manage children with neuro‐disabilities. Further research is therefore needed to understand needs to nutrition sector staff and develop appropriate training.

Acute malnutrition is generally managed through community management (CMAM) whereby children identified as wasted are provided with “ready to use therapeutic food” (RUTF), a high‐calorie peanut paste (Collins et al., 2006). These nutrition services are operated in the community with simple protocols; however, any child with a “complication” should be referred to an inpatient facility. Community implementers need to be better equipped with the knowledge to assess and refer children with CP. There may be an argument for blanket referral of all children with CP to nutrition services (Polack et al., 2018). These services need to be aware that deviation from standard protocols will likely be required when managing these cases, such as recognizing that children with CP may need to consume RUTF that has been modified in its consistency or to be provided with non‐peanut‐based RUTFs, which are less harmful to the lungs, if aspirated (Adams, 2009).

## 5 RECOMMENDATIONS

Caregiver training interventions including specific training on feeding can improve mealtimes for both children and caregivers. However, we recommend that future training programs include a greater emphasis on the feeding component, given the high prevalence of malnutrition in this population. Specifically, greater emphasis should be placed on positioning, further encouragement for self‐feeding, as well as improving dietary diversity. Involvement of other family members in the training program is also essential, especially in addressing physical exhaustion of caregivers and changing attitudes toward self‐feeding and associated spillages. The need to seek additional nutritional support should also be stressed to caregivers. While effective nutrition interventions are still lacking in settings relying on oral feeding, the improved utilization and effectiveness of mainstream therapeutic feeding programs are recommended. Staff at therapeutic feeding programs need guidance on the treatment of malnutrition in children with CP and other neuro‐disabilities, including the likely need to deviate from standard CMAM protocols.

## 6 LIMITATIONS OF STUDY

This subsample of caregivers was only recruited in half of the intervention districts and therefore may not be representative of all views. In addition, there was a small difference in the sample size from baseline to end line, although the majority of caregivers (11) participated in both. Another limitation could be that the interviewers may have been perceived as associated with the training program team, and despite stressing in the introduction that they were neutral, this may have affected the participant's desire to feedback honestly about the training program or during feeding observations. Lastly, as in other nutritional studies of children with CP, anthropometry can be inaccurate due to arm muscle wasting and inability to stand for height measurements. However, this study used innovative anthropometric methods to perform nutritional assessment on as many of the sample as possible, including use of knee height to estimate standing height. It is hoped that this will inspire other nutrition studies to do the same (Lelijveld & Kerac, 2017).

## 7 CONCLUSION

The findings of this study offer insight into mealtime challenges encountered by children with CP and their caregivers in Ghana, which are largely similar to those reported in studies from other countries. The results suggest that caregiver training can alleviate some of the difficulties faced by carers in relation to feeding their child with CP, and some of the stress associated with these. However, as in other studies where intervention involved caregiver training alone, there was no significant improvement in the children's anthropometric nutritional status. Besides attempts to further improve caregiver training, interventions need to go beyond this to address malnutrition. Better utilization and effectiveness of mainstream therapeutic feeding programs are necessary, including equipping these services to deviate from standard admission criteria and management protocols where necessary.

## ACKNOWLEDGMENTS

The authors would like to firstly thank the families who gave their time to take part in the study. We would also like to acknowledge the dedicated contribution of the study team in Ghana as well as other advisors across the globe, especially Sandra Carsamer, David O'Banion, Jedidia Abanga, Anthony Adoboe, John Agana, and Kabiru Abass. Lastly, we thank the Christian Blind Mission (CBM) who generously funded the creation and implementation of the “Getting to Know Cerebral Palsy” Training Programme.

## CONFLICT OF INTEREST

Authors declare no conflict of interest.

## 8 ETHICAL STATEMENT

The study's protocols and procedures were ethically reviewed and approved by two recognized ethical bodies: Noguchi Memorial Institute for Medical Research in Ghana (NMIMR‐IRB CPN 053/14‐15) and The London School of Hygiene and Tropical Medicine (LSHTM ref: 8905;10147;10880). Informed written or thumb‐printed consent was obtained from each participant in advance of the research.

## Supporting information


** **
