# Supplementary material for: Improving nutritional status of children with Cerebral palsy: a qualitative study of caregiver experiences and community‐based training in Ghana
Source: Food Sci Nutr. 2018 Nov 15;7(1):35–43. doi: 10.1002/fsn3.788 (PMC6341142; doi:10.1002/fsn3.788)
Supplement: Supplementary file 1 [file FSN3-7-35-s001.docx]

**Online Annex Figure 1:** qualitative coding tree depicting study themes and subthemes


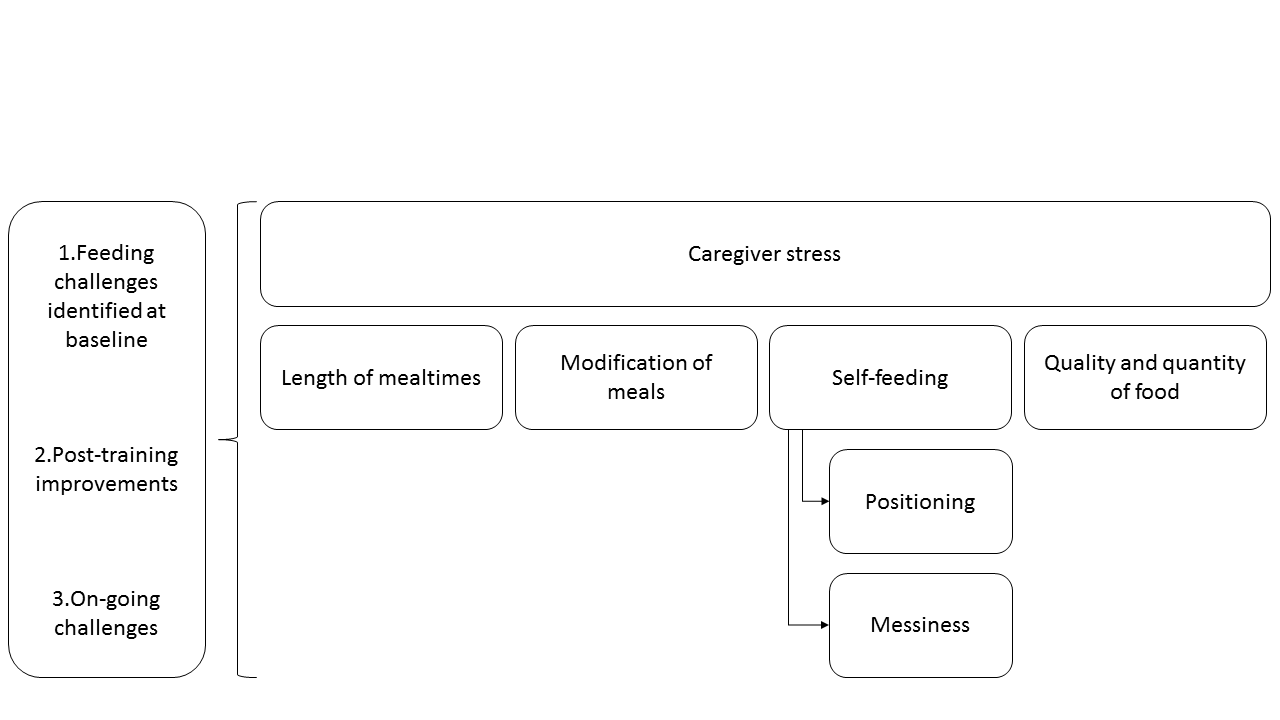


**Online Annex 2:** Table of individual nutrition status for children in the study at time of baseline and endline interviews

| **ID number** | **Baseline** | | | | **Endline** | | | |
| --- | --- | --- | --- | --- | --- | --- | --- | --- |
|  | **Weight for height**  **z-score** | **Height for age**  **z-score** | **MUAC (mm)** | **Nutritional status** | **Weight for height**  **z-score** | **Height for age**  **z-score** | **MUAC (mm)** | **Nutritional status** |
| C1 | -6.52 | -4.52 | 115 | Severely stunted and wasted | -1.36 | -5.20 | 123 | Severely stunted |
| C2 | - | -2.71 | - | Moderately stunted | - | - | - | Unknown |
| C3 | 1.40 | -2.59 | 180 | Moderately stunted | -1.36 | -4.59 | - | Severely stunted |
| C4 | -2.97 | -2.07 | - | Moderately stunted and wasted | -3.97 | -3.96 | - | Severely stunted and wasted |
| C5 | -3.54 | -4.45 | - | Severely stunted and wasted | -5.35 | -4.46 | - | Severely stunted and wasted |
| C6 | -3.03 | -1.22 | 131 | Severely wasted | -2.44 | -3.15 | 149 | Severely stunted and wasted |
| C7 | -3.78 | -0.36 | 138 | Severely wasted | -3.41 | -1.27 | 119 | Severely wasted |
| C8 | -0.48 | -2.12 | - | Moderately stunted | - | - | - | Unknown |
| C9 | 0.14 | -2.00 | 180 | Moderately stunted | -1.02 | -1.95 | 175 | Normal |
| C10 | -4.084 | -1.301 | 121 | Severely wasted | -2.10 | -1.71 | - | Moderately wasted |
| C11a | -3.716 | -1.118 | 139 | Severely wasted | -5.31 | -4.94 | 105 | Severely stunted and wasted |
| C11b | -2.50^‡^ | -2.50^‡^ | - | Moderately stunted and wasted | -2.74 | -2.75 | - | Moderately stunted and wasted |
| C12 | -3.297 | -0.941 | 140 | Severely wasted | -3.13 | -1.85 | 140 | Severely wasted |
| C13 | -1.72 | -1.968 | 155 | Normal | -0.73 | -0.20 | 155 | Normal |
| C14 | - | - | 168 | Normal | -0.07 | -0.31 |  | Normal |
| C15 | - | - | 151 | Normal | -1.75 | -1.77 | 161 | Normal |
| C16 | - | - | 176 | Normal | 0.50 | -1.50 | 214 | Normal |
| C17 | -0.48 | -2.12 | 164 | Moderately stunted | -1.52 | -2.34 |  | Moderately stunted |

MUAC= mid-upper arm circumference. ‡ indicates that exact measure not recorded: only between -2.0 and -3.0 known. MUAC and weight-for-height z-score only measured for children 6-59 months, hence some missing data.
